# Vasculocentric Axonal NfH in Small Vessel Disease

**DOI:** 10.1093/jnen/nlab134

**Published:** 2022-01-27

**Authors:** Adam Anad, Miriam K Barker, Jessica A Katanga, Konstantinos Arfanakis, Leslie R Bridges, Margaret M Esiri, Jeremy D Isaacs, Sonja Prpar Mihevc, Anthony C Pereira, Julie A Schneider, Atticus H Hainsworth

**Affiliations:** From the Molecular and Clinical Sciences Research Institute, St George’s University of London, London, UK (AA, MKB, JAK, LRB, JDI, ACP, AHH); Rush Alzheimer’s Disease Center, Rush University Medical Center, Chicago, Illinois, USA (KA, JAS); From the Molecular and Clinical Sciences Research Institute, St George’s University of London, London, UK (AA, MKB, JAK, LRB, JDI, ACP, AHH); Rush Alzheimer’s Disease Center, Rush University Medical Center, Chicago, Illinois, USA (KA, JAS); Department of Biomedical Engineering, Illinois Institute of Technology, Chicago, Illinois, USA (KA); From the Molecular and Clinical Sciences Research Institute, St George’s University of London, London, UK (AA, MKB, JAK, LRB, JDI, ACP, AHH); Department of Cellular Pathology, St George’s University Hospitals NHS Foundation Trust, London, UK (LRB); Nuffield Department of Clinical Neurosciences, Oxford University, Oxford, UK (MME); From the Molecular and Clinical Sciences Research Institute, St George’s University of London, London, UK (AA, MKB, JAK, LRB, JDI, ACP, AHH); Department of Neurology, St George’s University Hospitals NHS Foundation Trust, London, UK (JDI, ACP, AHH); Institute for Preclinical Sciences, Veterinary Faculty, University of Ljubljana, Ljubljana, Slovenia (SPM); From the Molecular and Clinical Sciences Research Institute, St George’s University of London, London, UK (AA, MKB, JAK, LRB, JDI, ACP, AHH); Department of Neurology, St George’s University Hospitals NHS Foundation Trust, London, UK (JDI, ACP, AHH); From the Molecular and Clinical Sciences Research Institute, St George’s University of London, London, UK (AA, MKB, JAK, LRB, JDI, ACP, AHH); From the Molecular and Clinical Sciences Research Institute, St George’s University of London, London, UK (AA, MKB, JAK, LRB, JDI, ACP, AHH); Department of Neurology, St George’s University Hospitals NHS Foundation Trust, London, UK (JDI, ACP, AHH)

**Keywords:** Alzheimer disease, Arteriolosclerosis, Brain aging, Neurofilaments, Small vessel disease, Vascular cognitive impairment

## Abstract

Cerebral small vessel disease (SVD) causes lacunar stroke and vascular cognitive impairment in older people. The pathogenic pathways from vessel pathology to parenchymal damage in SVD are unknown. Neurofilaments are axonal structural proteins. Neurofilament-light (NfL) is an emerging biomarker for neurological disease. Here, we examined the high molecular weight form neurofilament-heavy (NfH) and quantified a characteristic pattern of peri-arterial (vasculocentric) NfH labeling. Subcortical frontal and parietal white matter from young adult controls, aged controls, and older people with SVD or severe Alzheimer disease (n = 52) was immunohistochemically labeled for hyperphosphorylated NfH (pNfH). The extent of pNfH immunolabeling and the degree of vasculocentric axonal pNfH were quantified. Axonal pNfH immunolabeling was sparse in young adults but a common finding in older persons (controls, SVD, or AD). Axonal pNfH was often markedly concentrated around small penetrating arteries. This vasculocentric feature was more common in older people with SVD than in those with severe AD (p = 0.004). We conclude that axonal pNfH is a feature of subcortical white matter in aged brains. Vasculocentric axonal pNfH is a novel parenchymal lesion that is co-located with SVD arteriopathy and could be a consequence of vessel pathology.

## INTRODUCTION

Cerebral small vessel disease (SVD) is a common vascular lesion in brains of older people (1–3). SVD affects deep penetrating arteries and is characterized by concentric fibrotic thickening of the arterial wall, with depletion of myocytes, and sparing of endothelia (4–6). SVD is associated with lacunar stroke (3, 4, 7), diffuse white matter lesions (seen as white matter hyperintensities on T2-weighted MRI) (8, 9), and subcortical microbleeds (10). SVD is a common cause of vascular cognitive impairment (3, 11, 12). In SVD, the molecular mechanisms linking small artery changes with parenchymal lesions are poorly understood. As a result, there are few established molecular biomarkers for SVD, and no targeted therapies.

Neurofilament proteins maintain the shape and mechanical strength of nerve axons and mediate axonal transport (13–16). There are 3 isoforms: neurofilament-light (NfL, molecular weight: 68 kDa), medium (NfM, 150 kDa), and heavy (NfH, 210–220 kDa, depending on the degree of phosphorylation) (14, 16, 17). NfL assayed in cerebrospinal fluid (CSF) and blood is becoming established as a quantitative biomarker of neurodegenerative damage (15, 18–20).

The high molecular weight form of NfH has a C-terminal domain containing multiple Lys-Ser-Pro (KSP) motifs, which are potential phosphorylation sites (13, 14, 16). Antibodies with high specificity for NfH (relative to other neurofilament types) have been developed and are the basis of NfH detection kits for screening biofluids. The SMI35 (17, 21) and RT97 (22) monoclonal antibodies are selective for hyper-phosphorylated NfH (pNfH). High blood levels of pNfH have been reported in patients with neurological conditions characterized by acute neuronal damage, enabling early detection. These conditions include acute optic neuritis (23), acute stroke (24), traumatic brain injury (25), multiple sclerosis (17, 26), amyotrophic lateral sclerosis (ALS) (27), and neuromyelitis optica (28), as recently reviewed (15).

Here, we investigated the pattern of pNfH immunolabeling in brains of older people with moderate-severe SVD and compared this with older people with severe AD pathology, older people without brain pathology, and young healthy adults.

## MATERIALS AND METHODS

### Research Involving Biological Material and Data From Human Participants

Ethical approval for the use of human brain tissue in this study was provided by the UK National Research Ethics Service (East Midlands-Derby research ethics committee, Ref#12/EM/0028). The study was performed in accordance with the ethical standards as laid down in the 1964 Declaration of Helsinki and its later amendments. Most of the human tissue samples were from the Oxford Brain Bank (REC approval#15/SC/0639). Three cases were from Rush Alzheimer’s Disease Center (ID numbers 32, 48, and 52 in the Table). Written informed consent was received from participants or their next-of-kin prior to inclusion in the study.

**Table.** Demographics and pNFH Quantitation for the Cases Studied

Study ID #	Group (YC, AC, SVD, or AD)	Sex (F/M)	Age at Death (years)	Postmortem Interval (hours)	Mean pNFH-Positive Area Fraction (range: 0–1.0)*	Proportion of Small Arterial Vessels With Vasculocentric Axonal pNFH (range: 0–1.0)^†^	Small Artery Density (vessels/mm^2^)^‡^
1	YC	M	20	NR	0.006	0.000	0.095
2	YC	F	26	72	0.292	0.000	0.400
3	YC	M	36	41	0.070	0.286	0.147
4	YC	M	41	24	0.003	0.081	0.097
5	YC	F	42	48	0.026	0.267	0.110
6	YC	M	45	48	0.006	0.125	0.063
7	YC	F	48	48	0.757	0.744	0.542
8	YC	M	51	24	0.064	0.400	0.128
9	YC	M	56	48	0.091	0.395	0.287
10	YC	M	56	16	0.076	0.440	0.089
11	YC	M	59	24	0.051	0.416	0.211
**YC Group, Mean (SD)**		**3F/8M**	**43.6 (12.5)**	**39.3 (17.1)**	**0.131 (0.223)**	**0.287 (0.225)**	**0.197 (0.153)**
12	AC	M	69	63	0.116	0.688	0.096
13	AC	M	79	28	0.010	0.156	0.115
14	AC	F	80	21	0.006	0.391	0.095
15	AC	M	83	88	0.011	0.571	0.135
16	AC	M	85	56	0.063	0.474	0.357
17	AC	M	86	3	0.045	0.353	0.077
18	AC	F	88	115	0.518	0.500	0.299
19	AC	M	91	3	0.004	0.158	0.109
20	AC	F	91	4	0.050	0.536	0.165
21	AC	M	93	114	0.009	0.588	0.089
22	AC	M	97	6	0.057	0.889	0.044
**AC Group, Mean (SD)**		**3F/8M**	**85.6 (7.8)**	**45.5 (44.2)**	**0.081 (0.149)**	**0.482 (0.216)**	**0.144 (0.097)**
23	SVD	M	62	96	0.029	0.318	0.066
24	SVD	M	65	144	0.048	0.622	0.113
25	SVD	M	71	49	0.042	0.324	0.116
26	SVD	M	76	29	0.150	0.913	0.237
27	SVD	M	77	75	0.050	0.769	0.045
28	SVD	M	78	141	0.049	0.679	0.130
29	SVD	M	78	7	0.004	0.063	0.067
30	SVD	M	80	84	0.028	0.583	0.099
31	SVD	M	82	56	0.043	0.538	0.129
32	SVD	M	82	6	0.063	0.842	0.143
33	SVD	F	83	45	0.224	0.800	0.122
34	SVD	M	83	41	0.745	0.778	0.172
35	SVD	F	83	NR	0.295	0.649	0.059
36	SVD	F	85	62	0.024	0.488	0.229
37	SVD	M	93	8	0.367	0.125	0.058
38	SVD	M	93	36	0.279	0.464	0.102
39	SVD	F	95	96	0.065	0.529	0.170
**SVD Group, Mean (SD)**		**4F/12M**	**80.3 (9.2)**	**64.6 (41.8)**	**0.152 (0.195)**	**0.540 (0.239)**	**0.120 (0.058)**
40	AD	F	66	140	0.474	0.364	0.090
41	AD	M	71	41	0.816	0.000	0.232
42	AD	F	72	48	0.103	0.333	0.060
43	AD	M	73	6	0.066	0.000	0.122
44	AD	F	76	NR	0.382	0.625	0.224
45	AD	F	81	56	0.084	0.391	0.076
46	AD	M	81	69	0.723	0.000	0.073
47	AD	M	89	52	0.063	0.263	0.069
48	AD	M	90	21	0.157	0.931	0.172
49	AD	F	90	33	0.176	0.552	0.097
50	AD	M	91	6	0.579	0.000	1.399
51	AD	M	94	60	0.033	0.458	0.118
52	AD	F	97	10	0.264	0.882	0.106
**AD Group, Mean (SD)**		**5F/6M**	**80.4 (9.5)**	**51.1 (37.8)**	**0.318 (0.290)**	**0.271 (0.237)**	**0.233 (0.391)**

AC, aged control; AD, Alzheimer disease; NR, not recorded; SVD, small vessel disease; YC, young control.

* Mean pNFH-positive area fraction (range: 0–1.0) is a measure of the extent of immunolabeling. It was calculated for each image as the ratio of (number of pNfH-positive pixels)/(total pixels in the image) and the average across all images is reported for each case.

^†^Proportion of small arterial vessels with vasculocentric axonal pNFH (range: 0–1.0) was determined for each case by examining all small vessels of arterial appearance with outer diameter 100–300 μm, in subcortical white matter. It was calculated as the ratio (number of vessels with vasculocentric pNfH labeling)/(total number of vessels).

^‡^Small artery density (vessels/mm^2^) was calculated as (total number of small vessels of arterial appearance with outer diameter 100–300 μm, in subcortical white matter)/(total area analyzed in mm^2^).

### Neuropathological Assessment of SVD

Assignment to neuropathological SVD was based on microscopic examination of hematoxylin and eosin sections by registered neuropathologists (M.M. Esiri or Dr. Catherine Joachim) blind to the assessment of pNfH immunolabeling, which was performed subsequently. SVD was defined by vasculopathy-oriented criteria, as in our previous studies (6, 11, 29). These included hyaline thickening of arteriolar walls; widened perivascular spaces; and parenchymal changes considered to result from SVD (perivascular pallor of myelin staining, loosening with attenuation of nerve fibers with gliosis in white matter or loss of nerve cells and gliosis in deep gray matter) in one or more sections (6, 11, 29). For SVD cases, we selected those with moderate or severe SVD according to these criteria, and with minimal AD pathology (Braak stage 0–II).

### Neuropathological Assessment of AD

Neuropathological assessment according to CERAD, NIA-Reagan criteria, and Braak staging were documented for all older cases, based on routine immunolabeling for Aβ (4G8 antibody) and p-tau (AT8 antibody) as well as hematoxylin and eosin and Luxol fast blue/cresyl violet stains (30, 31). Neurofibrillary tangle (Braak stage) and amyloid pathology (CERAD rating scheme) were assessed by an experienced diagnostic neuropathologist (30). For severe AD cases, we selected those with Braak stage V–VI and with absent or mild SVD.

### Human Brain Immunohistochemistry

Frontal and parietal cortical tissue blocks, including subcortical white matter, were examined. Formalin-fixed paraffin-embedded sections were immunohistochemically labeled as described previously (29, 32). Endogenous peroxidase activity was blocked by exposure to H_2_O_2_ (3% v/v, aqueous solution) for 8 minutes. After high-pressure heat-induced antigen retrieval (30 seconds, 125°C, in pH 7.8 Tris-citrate buffer), nonspecific binding was blocked with phosphate-buffered saline containing 0.1% v/v Triton-X100 and 3% (w/v) bovine serum albumin (PBT-BSA) for 60 minutes at room temperature. Sections were then exposed to primary antibodies at 4°C overnight.

Primary antibodies were diluted on the day of use in PBT-BSA. Primary antibodies were the following. Hyper-phosphorylated NfH (mouse monoclonal, clone SMI35; diluted 1:100 000; Covance, Dedham, MA) Hyper-phosphorylated NfH (mouse monoclonal RT97; diluted 1:30 000; BioRad-SeroTec, Watford, UK). Neurofilament (mouse monoclonal, clone 2F11; diluted 1:3000; Dako, Carpinteria, CA). The SMI35 IgG_1_ monoclonal antibody (originally named clone #03-44) was raised against rat hypothalamic homogenate and has undergone extensive characterization by the originators (21, 33, 34) and by other authors (17, 35). The RT97 IgG_1_ monoclonal antibody was raised against a rat brain protein Triton-X100-insoluble fraction, produced in a different research laboratory (22) and characterized by different groups (22, 36).

Primary antibody labeling was visualized using a peroxidase-conjugated secondary reagent and diaminobenzidine (DAB) chromagen (Envision^®^ kit, K4065, Dako-Agilent, Ely, UK) then counterstained with Mayer’s hematoxylin. As a negative control, neighboring sections were treated identically with an irrelevant primary antibody (mouse monoclonal IgG_1_ against HPV, clone K1H8, diluted 1:600; Dako, Glostrup, Denmark).

### Quantifying pNfH Immunolabeling in Digital Pathology

Labeled sections were scanned using a digital slide scanner under 20× objective lens (Hamamatsu NanoZoomer 2.0-RS slide scanner) within St George’s Imaging Resource Facility. Scanned sections were stored as large digital files (typically 1 Gb) in the manufacturer’s image format, NanoZoomer Digital Pathology Image (.ndpi). To quantify features of immunolabeling, ndpi files were viewed using Hamamatsu viewing software NDP View 2. Scanning of sections, harvesting of TIFF files, and all image analyses were performed blind to clinical data. Quantitation of all cases was performed by one author (A.A.). For a subset of cases (n = 23), quantitation of the vasculocentric fraction of pNfH labeling was repeated by a second blinded observer (S.M.).

In order to calculate the extent of pNfH immunolabeling, a grid was superimposed onto the scanned section and smaller images were sampled in a systematic manner. Images were sampled in TIFF format at the intersections of gridlines within subcortical white matter at 10× magnification. At least 5 fields of view were obtained for each section. The field of view at the intersection was not sampled if any of the following were present: the presence of gray matter; the presence of periventricular ependymal lining; vessels greater than 300 μm in outer diameter; unusually large perivascular spaces; poor tissue quality, such as tearing or other artifacts. TIFF images were imported into FIJI-ImageJ software (National Institute for Health). Using the H-DAB vector for Color Deconvolution-2 plugin for ImageJ (https://blog.bham.ac.uk/intellimic/g-landini-software/colour-deconvolution-2/), a map of DAB labeled pixels was obtained in 8-bit grayscale format. For each TIFF image, DAB-positive pixels were identified using the inbuilt threshold detection algorithm with Renyi-Entropy window. The extent of labeling was defined as the area fraction, calculated as the ratio of (number of DAB-positive pixels/total pixels in the image), and the average across all TIFFs was reported for each case.

In order to determine the vasculocentic fraction of pNfH labeling, scanned sections were viewed in Hamamatsu NDP-View2 viewing software at 10× magnification. Sections were assessed for the presence of small vessels of arterial appearance with outer diameter of 100–300 μm. Sections were viewed systematically from the top left corner, in a “lawnmower” fashion. Only clearly recognizable white matter was included in the area of analysis. Any areas of poor tissue quality or clear artifact (tears, folds, bubbles) were omitted. Each vessel was assessed qualitatively for the presence of vasculocentric axonal pNfH labeling. The total area of white matter analyzed was recorded, using NDP-View2 software. For each section, the ratio of the number of vessels with vasculocentric pNfH labeling to the total number of vessels was reported. The vessel density was reported as (total number of included vessels/total area analyzed in mm^2^).

Agreement between the 2 independent observers (A.A., S.P.M.) was assessed in terms of the Pearson correlation coefficient R and the mean difference and Bland-Altmann limits of agreement (mean difference ± [1.96 × standard deviation of the difference]). For the total number of vessels per section, the number of vessels with vasculocentric pNfH labeling, and the proportion of vessels with vasculocentric pNfH labeling: R was 0.702, 0.674, and 0.719, respectively; the mean difference and limits of agreement were 4.0 (−9.3, 17.3), 1.5 (−9.5, 12.5), and −0.088 (−0.541, 0.364), respectively.

### Statistics

Statistical testing was performed in SPSS (v.26). Between-group differences were tested using Kruskal-Wallis tests, with Dunn post hoc testing. No post hoc corrections were applied. P < 0.05 was considered significant.

## RESULTS

We examined subcortical white matter from 52 brains. These included 11 younger adults without brain pathology (mean [SD] age: 43.6 [12.5] years), 11 older people without significant brain pathology (85.6 [7.8] years), 17 older people with moderate-severe SVD (80.4 [9.0] years), and 13 older people with severe AD (82.4 [10.1] years). Demographic details are listed in the Table.

### Characterization of pNfH Immunolabeling in Subcortical White Matter

**FIGURE 1.**
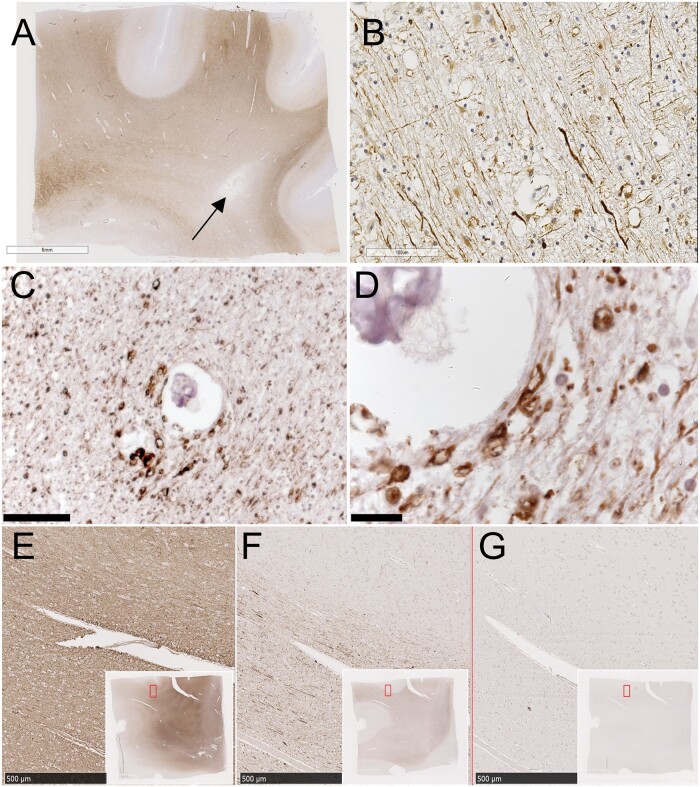
Hyper-phosphorylated NfH immunolabeling in older people. **(A)** Low-magnification view of a typical section immunolabeled with the SMI35 antibody (visualized with DAB chromogen, brown). Nuclear chromatin is counterstained with hematoxylin (blue). The donor was an older person with moderate-severe SVD. A small infarcted lesion is visible (arrow). The section is taken from frontal cortex including subcortical white matter. **(B)** Higher-magnification view of white matter within the same section shows strongly positive axons in transverse section. **(C)** Subcortical white matter in a different case shows axons in cross section. **(D)** Higher-magnification view shows axonal bulbs around a small arterial vessel. **(E–G)** Neighboring sections immunolabeled for pan-selective neurofilament monoclonal antibody **(E)**, pNfH selective antibody SMI35 **(F)**, or irrelevant primary antibody HPV **(G)**. Insets show the section maps, with region of interest outlined in red. Scale bars: **A** = 6 mm, **B, C** = 100 μm, **D** = 20 μm, **E–G** = 500 μm.

In brains of older people, across all groups, there was a characteristic labeling pattern. In subcortical white matter, axons were strongly positive for pNfH (Fig.  1A, B). Frequently, swollen structures that appeared to be axonal bulbs were pNfH-positive and clearly visible (Fig.  1C, D). The axonal bulbs were a feature in the majority of older cases, at least 60%. They varied in size, some appearing to have an unlabeled interior, possibly a cavity. In overlying gray matter, pNfH labeling was sparse (Fig.  1A, F). In focal, cavitated lesions, assumed to be microinfarcts, axonal labeling with pNfH was absent or sparse (Fig.  1A).

In neighboring sections labeled with a pan-selective neurofilament antibody, the density of labeled axons was clearly much greater than with pNfH (Fig.  1E, F). As a result, in pan-neurofilament labeled material, the swollen axonal bulbs, though positive, were not readily visible on qualitative inspection. Axonal labeling was absent in negative control sections treated with an irrelevant monoclonal antibody (anti-HPV, Fig.  1G).

### Vasculocentric pNfH Labeling

**FIGURE 2.**
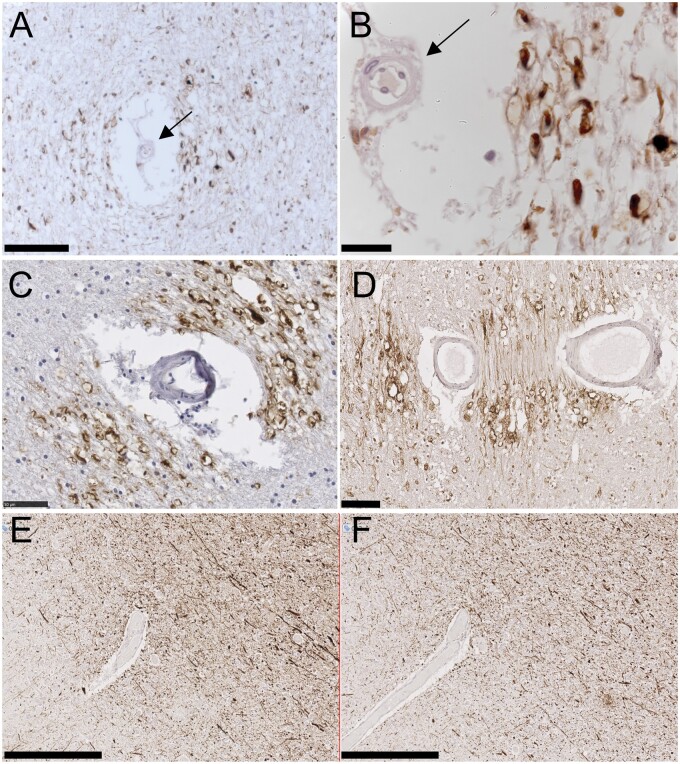
Vasculocentric axonal pNfH labeling in older people. **(A, B)** pNfH-positive swollen axons and axonal bulbs around a small artery. The vessel is identified as an artery (arrows) by the wide perivascular space, the wall thickness relative to lumen diameter, and the mural smooth muscle cell. **(C, D)** Two examples of a clear vectorial pattern of axonal pNfH labeling around small arteries. The 2 lumina in panel **(D)** are likely the same vessel. **(E, F)** Neighboring sections immunolabeled with 2 different monoclonal antibodies raised against hyper-phosphorylated NfH: SMI35 **(E)** and RT97 **(F)**. Scale bars: **A**, **D** = 100 μm, **B** = 20 μm, **C** = 50 μm, **E, F** = 250 μm.

In brains of older people, we frequently observed a “vasculocentric” pattern of pNfH labeling. Axons that were strongly positive for pNfH (labeled with the SMI35 monoclonal antibody), including noticeable axonal bulbs, were clustered around a small blood vessel with arterial appearance (examples in Fig.  2). Vessel-centered pNfH labeling was not seen around vessels of venous appearance (not shown). In some examples of vasculocentric axonal pNfH labeling, the distribution of axonal labeling was not symmetrical around the vessel but displayed a vectorial orientation, examples in Figure  2C, D. This may have resulted from the orientation of the blood vessel within the tissue. Serial sections would be required to confirm this. A different monoclonal antibody RT97, also selective for hyper-phosphorylated NfH, produced a labeling pattern qualitatively similar to that of SMI35 (Fig.  2E, F). This included axonal bulbs and the vasculocentric orientation around small arteries.

### Qualitative Observations in Young Adults, and in Older Adults With SVD or AD

**FIGURE 3.**
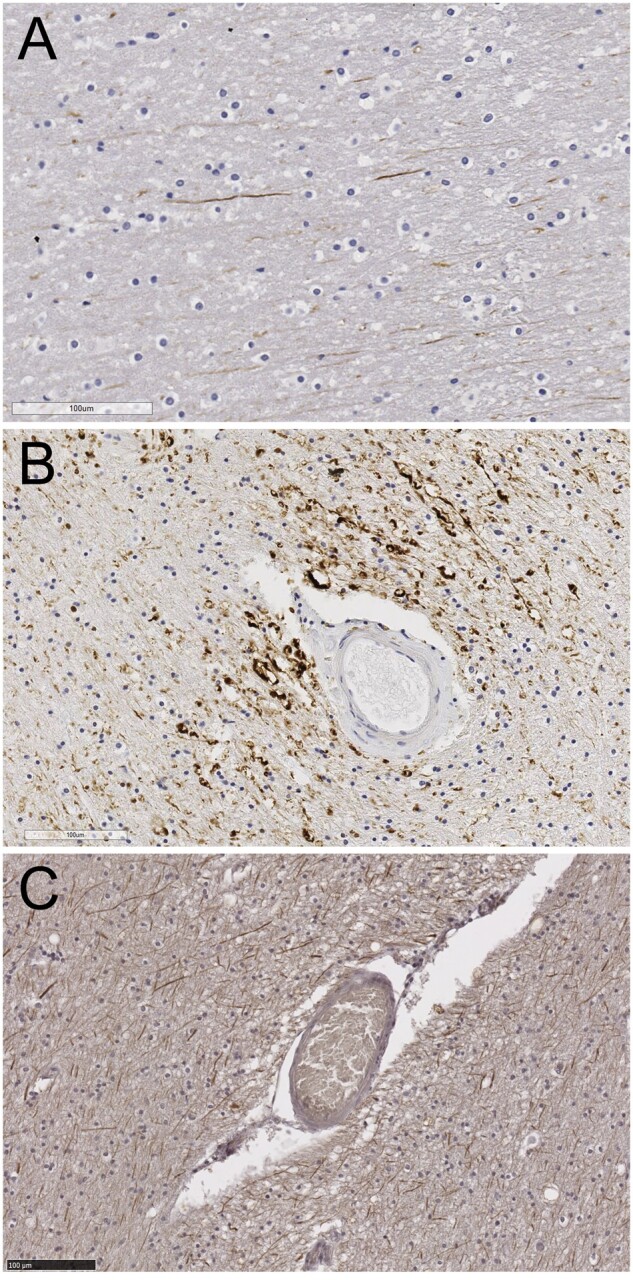
Hyper-phosphorylated NfH immunolabeling in a young adult and older persons with SVD or with AD. **(A)** Young adult (male, age 20). Sparse axonal pNfH labeling is seen in subcortical white matter. **(B)** Older person with small vessel disease. The small arterial vessel exhibits fibrotic thickening, characteristic of SVD. Strongly positive axons and axonal bulbs are seen around the vessel. **(C)** Older person with severe Alzheimer disease pathology (Braak stage VI). Axonal labeling is widespread but lacks vasculocentric orientation. Scale bars: 100 μm.

In tissue from similar regions in healthy young adults, pNfH labeling was absent or sparse (example in Fig.  3A). In subcortical white matter, a few pNfH-positive axons could be found. Axonal bulbs were not seen. In older people with a neuropathological diagnosis of SVD, the pNfH labeling configuration of strongly positive axons, positive axonal bulbs and vasculocentric axonal labeling (Fig.  3B) was evident in white matter from all cases examined (n = 41, Table). In older people with severe AD pathology (Braak stage V–VI) the pNfH labeling configuration was different with abundant strongly positive axons and some axonal bulb labeling, but vasculocentric axonal labeling was less evident (Fig.  3C).

**FIGURE 4.**
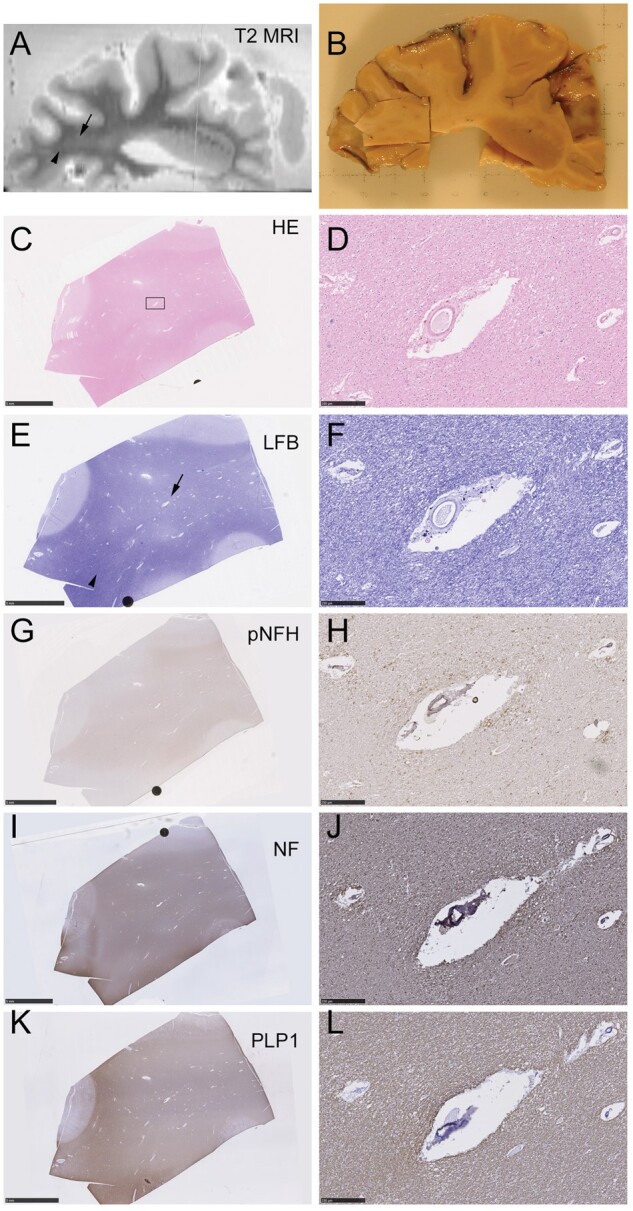
Ex vivo MRI-directed sampling of T2 hyperintense subcortical white matter. RADC-derived tissue stained for hematoxylin and eosin (H&E) and Luxol fast blue (LFB) and immunolabeled for pNfH, pan-neurofilament (NF), and myelin (PLP1). **(A)** Ex vivo T2-weighted MRI of a 1-cm-thick slice of fixed brain tissue. A region of WMH is visible (arrow), adjacent to an area of nonhyperintense normal-appearing white matter (arrowhead). **(B)** At gross pathology, the sampled tissue block contains the region of WMH seen in panel **(A)**. **(C, D)** H&E staining of the sampled tissue block is shown in panel **(B)**. The low magnification view in panel **(C)** shows the region (rectangle) that is magnified in the right-hand panels, containing a landmark blood vessel. **(E, F)** LFB-stained section exhibits white matter pallor (arrow) in region identified as WMH in the T2-weighted scan in panel **(A)**. This contrasts with the more densely stained area (arrowhead) identified as normal appearing white matter in panel **(A)**. **(G, H)** Immunolabeling for pNfH (SMI35 antibody) is not noticeably more pronounced in the area of WMH, though high magnification confirms the vasculocentric axonal pattern **(H)**. **(I, J)** Axonal neurofilament (pan-neurofilament antibody) shows no marked loss of positivity in the WMH region. **(K, L)** Myelin immunolabeling (PLP1 antibody) resembles LFB in terms of pallor within the WMH region **(K)**. Scale bars: 5 mm in low-magnification images, 250 μm in high-magnification images.

For 3 cases (ID numbers 32, 48, and 52 in the Table), ex vivo T2-weighted MRI scans were available to guide tissue sampling (example in Fig.  4A, B). In subcortical white matter hyperintensities (WMH), the staining with hematoxylin and eosin and Luxol fast blue (LFB) was clearly pale, relative to neighboring nonhyperintense white matter (Fig.  4C–F). In these regions of pallor, pNfH immunolabeling did not appear extensive or strongly positive, on examination (Fig.  4G, H). The extent of labeling with a pan-neurofilament or myelin marker (PLP1) did not appear noticeably different, relative to nonhyperintense white matter (Fig.  4I–L) though the overall intensity of PLP1 appeared pale in WMH, corresponding to pallor in LFB-stained neighboring sections (Fig.  4E, K).

### Semiquantitative Analysis of pNfH Immunolabeling

**FIGURE 5.**
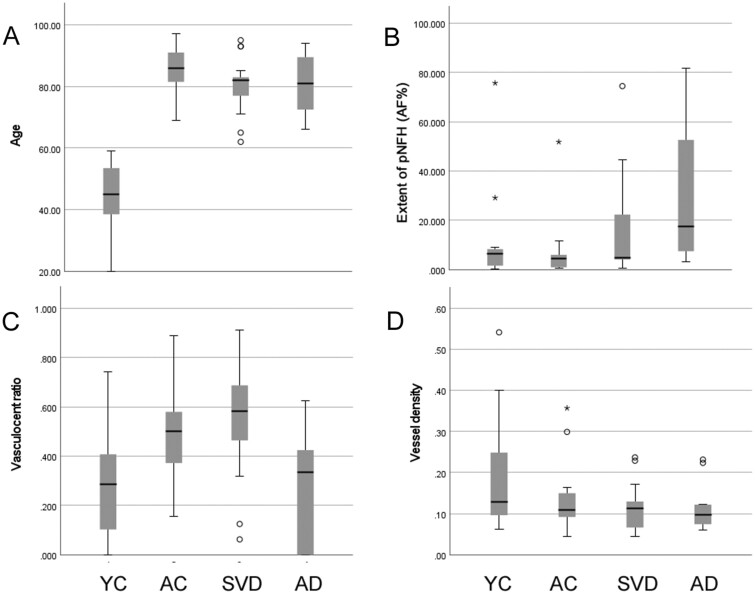
Comparison of age at death, extent of pNfH immunolabeling, pNfH vasculocentric ratio, and vessel density. **(A)** Age at death (years). **(B)** Extent of pNfH immunolabeling within subcortical white matter (percent area fraction, AF%). **(C)** The proportion of small arteries exhibiting a vasculocentric pattern of axonal pNfH labeling (vasculocentric ratio). **(D)** Density of small arterial vessels within sections of subcortical white matter (vessels/mm^2^). Data are shown for 4 groups: young adult controls (YC), aged controls (AC), older people with moderate-severe small vessel disease (SVD), and older people with severe AD pathology (AD). Box-whisker plots show the group median, interquartile range (IQR), and full range. Open circles show outliers >1.5 IQR from the end of a box. Asterisks show outliers >3 IQR from the end of a box.

In SMI35 immunolabeled sections, we analyzed 2 quantitative densitometric measures of pNfH labeling: first, the extent of immunolabeling within subcortical white matter (quantified as the area fraction); second, the proportion of small arteries with a vasculocentric pattern of axonal labeling (termed the vasculocentric ratio). We compared these across 4 groups: young adult controls, aged controls, older people with SVD, and older people with severe AD pathology. Comparing the 4 groups, a significant difference in terms of age was identified (p < 0.001, Kruskal-Wallis test; Fig.  5A) and post hoc testing indicated that the young controls differed from all other groups (all p < 0.001). Other between-group differences in age were not significant (p ≥ 0.236). The immunolabeled area fraction was generally low in young adults and aged control cases but showed a wide range among those with SVD or AD pathology (Fig.  5B). Across the 4 groups, there was a significant difference between groups (p = 0.025, Kruskal-Wallis test; Fig.  5) and post hoc testing indicated those with AD had higher area fraction than young or aged controls (p = 0.033 and p = 0.003, respectively). Considering the vasculocentric ratio, there was a considerable range within each of the 4 groups (Fig.  5C). There was a significant difference between groups (p = 0.007, Kruskal-Wallis test). Post hoc testing indicated that those with SVD had higher vasculocentric ratio than young controls, or those with AD (p = 0.006, 0.004, respectively). Those with AD did not reach significance relative to aged controls (p = 0.051). In terms of vessel density, no significant difference was detected across the 4 groups (p = 0.514, Kruskal-Wallis test; Fig.  5D).

## DISCUSSION

The main findings of this study are as follows. First, axonal pNfH immunolabeling is scarce in brains of young adults but a common finding in brains of older persons (controls, SVD, or AD). Second, pNfH labeling is much more focal than a pan-neurofilament labeling pattern, often markedly concentrated around small arteries. Third, this vasculocentric feature was more common in older people with moderate-severe SVD than in those with severe AD.

### Neurofilaments and the Aging Brain

The lack of pNfH labeling in young control brains, compared to its universal presence in aged brains, suggests that pNfH accumulation may be a feature of brain aging. The appearance of strongly positive axonal swellings and bulbs suggests that axonal pNfH may not reflect benign aging but may be related to axonal pathology. Elevated levels of pNfH in blood and CSF of individuals with diverse brain injury states (15, 24, 25) support the concept that pNfH is associated with brain pathology.

In common with other cytoplasmic proteins, NfH undergoes dynamic turnover of phosphate groups. The NfH protein contains 44 Ser or Thr residues that are targets for phosphorylation, with most Ser residues located in Lys-Ser-Pro (KSP) repeats of the C-terminal domain (37). Client kinases for NfH include Cdk5, GSK-3β, and cdc2 (16, 36–38). How pNfH is related to axonal damage is unknown. Robust axonal pNfH positivity has been reported by ourselves and others in a variety of different CNS disease states, including SVD, AD (this study), and multiple sclerosis (17). It seems unlikely that pNfH accumulation is causal in all these, but may be a downstream manifestation. We have not explored the possibility that axonal pNfH accumulation may be accompanied by retrograde pNfH labeling in neuronal perikarya and dendrites, as reported for focal stroke lesions (39).

### Neurofilaments in Relation to Dementia and SVD

NfL assayed in blood and CSF appears increasingly to be a quantitative biomarker of neurodegenerative disease (15, 18–20, 27, 40). Considering pNfH concentrations in CSF and blood as a possible marker for dementia, some but not all studies have demonstrated elevated pNfH in AD patients relative to controls (13, 35, 41), as recently reviewed (15). In patients with a clinical diagnosis of “dementia of vascular origin,” CSF concentrations of pNfH were significantly elevated relative to control subjects (13, 41).

Blood NfL concentration is associated with SVD. In both sporadic and genetic forms of SVD, blood NfL was elevated relative to controls, and strongly associated with MRI markers of SVD severity (42). MRI evidence of recent subcortical infarcts or high burden of white matter lesions (often used as a radiological indicator of SVD) was associated with high blood concentrations of NfL (43). Our neuropathology data suggest that pNfH in brain tissue may also be associated with SVD.

### Hyperphosphorylated NfH as a Biomarker for Axonal Damage

High levels of pNfH have been reported in CSF and blood of patients with neurological disease (15), including acute stroke (24), traumatic brain injury (25), multiple sclerosis (17), ALS (27), Friedrich’s ataxia (44), and genetic frontotemporal dementia (45). In a Phase III trial in traumatic brain injury, blood pNfH was used as a biomarker of injury severity and possible predictor of outcome (25). That study found that high blood pNfH levels were correlated with poor outcome at 3–4 days following traumatic injury (25).

Commercial assays for pNfH in blood are available, with high sensitivity and specificity, including ultra-sensitive Simoa platforms (14, 18, 35, 40). There is a poor correlation between blood levels of NfL and pNfH within subjects (27, 46). In a large, prospective observational study of patients with ALS, baseline blood concentrations of NfL but not pNfH predicted disease progression (27). In children with spinal muscular atrophy (SMA), blood levels of NfH may be a diagnostic marker for disease onset (47) though paradoxically blood levels declined in older children with chronic disease progression (47). A recent phase II trial in optic neuritis patients included blood NfH as an endpoint. There was a significant reduction in blood pNfH (but not blood NfL) after 3 months of treatment, relative to placebo (46). These various reports suggest that, despite their similar biological origin, pNfH appears to be a distinct biomarker from NfL.

### Vasculocentric pNfH in Subcortical White Matter

The vasculocentric pNfH labeling pattern, we observed was notable for being associated with the small arterial vessels that are central to SVD pathology. So far as we are aware this is the first report of a parenchymal lesion that is physically co-located with SVD arteriopathy. Other instances of vasculocentric axonal labeling have been reported. Axonal positivity for p-tau with a vascular orientation has been noted in chronic traumatic encephalopathy (CTE) (48–50). CTE is a neurodegenerative state characterized by excessive accumulation of p-tau (49) and perivascular or vasculocentric p-tau labeling was described (48, 50). In contrast to the pNfH pattern reported here, the vasculocentric p-tau in CTE appears to be located within gray matter (cortex, amygdala) (48, 50). Vasculocentric labeling has not been reported in brain material immunolabeled with other standard neurofilament markers, such as SMI32 (51).

We speculate that the observed vasculocentric pNfH pattern may be due to an aspect of SVD arterial pathology causing axonal injury in subcortical white matter. Several speculations can be made regarding the cause of vasculocentric axonal pNfH. First, it may be due to leakage of an axon-damaging blood component from the small artery lumen, across a defective blood-brain barrier (BBB). This appears unlikely as the vessel-centered pattern is generally restricted to arteries, while BBB permeability may occur in other compartments of the vascular tree (venules, veins) (29, 32). Second, dysfunction of perivascular fluid clearance may cause local axonal damage or toxicity (52, 53). Third, the pattern may reflect chronic physical trauma. The persistence of a pulse wave propagated into deep penetrating arteries is evidenced by 7T MRI data (54). Combined with widening Virchow-Robin spaces filled with perivascular fluid, pulsatile flow in a poorly distensible tube may inflict chronic repetitive stress on adjacent parenchymal tissue.

### Limitations

This report has several limitations. First, the cohort is small and affords relatively low statistical power. Validation in a larger, well-characterized brain cohort would be advantageous. Second, as with all postmortem neuropathological studies, we are assessing brain disease at an advanced stage.

### Conclusions

In conclusion, axonal pNfH is a feature of aged brains. Hyperphosphorylation of axonal NfH in subcortical white matter is frequently vasculocentric. This vessel-centered pattern is associated with SVD and may be a consequence of SVD arteriopathy.

## Notes

This study was supported by Alzheimer’s Society (UK) (PG146/151), Alzheimer’s Drug Discovery Foundation (Project Ref 20140901), Alzheimer’s Research UK (PPG2014A-8), and UK Medical Research Council (MR/T033371/1) to A.H.H.; National Institute of Neurological Disorders and Stroke (NINDS) (UH3NS100599, UF1NS100599) to K.A. and J.A.S.; European Regional Development Fund to S.P.M.

A.H.H. has received honoraria from Eli-Lilly and from NIA, he chairs the Vascular Cognitive Disorders PIA within ISTAART, and he leads the Dementias Platform UK Vascular Experimental Medicine group. J.D.I. has received conference expenses from Roche. His institution has received a speaker’s fee from Biogen and an advisory board fee from Roche. S.P.M. is an employee of Novartis. All other authors report no relevant conflicts of interest.
